# Fostering clinical practice in IR regardless of (sub) specialty status

**DOI:** 10.1186/s42155-023-00370-1

**Published:** 2023-04-06

**Authors:** Christoph A. Binkert

**Affiliations:** Clinic of Interventional Radiology and Vascular Surgery, Kantonsspital Winterthur, Winterthur, Switzerland

Charles Dotter is considered the founder of interventional radiology when he performed the first angioplasty in 1964. Since then interventional radiology has experienced a tremendous technical development. Unlike Charles Dotter who advocated direct patient care already at that time, most early interventional radiologists were “proceduralists”. They performed the procedure, but did not care for the patient before and after the procedure. The workflow was similar to diagnostic radiology: a referring physician orders an exam and the radiologist then performed the ordered intervention. After the procedure the patient went back to the referring physician for further care.

This way of practicing made the interventional radiologist invisible for patients. Despite performing the key part of the therapy, the patient often regarded the referring physician as the treating physicians. Therefore nearly no one is nowadays aware that coronary artery catheterization was initially widely performed by diagnostic radiologist. The switch to cardiology was not due to better skills, but because cardiologists cared about the patient. The disappearance of radiology from cardiac catheterization went nearly unnoticed because nobody knew who was actually doing it.

In the early 2000’s the same fate threatened to happen in the United States of America for endovascular treatment of peripheral vascular disease. The interventional radiology community realized that without clinical involvement the field of arterial interventions, and maybe other areas as well, would be lost to vascular surgery or cardiology or other disciplines. At that time a change in mindset happened. Interventional radiologists started to get involved in clinical responsibilities for their patients. Outpatient clinics were created to see and consult the patients before the procedure and for follow-up after the procedure. By doing so, the interventional radiologist became visible for the patient and also for the wider health care community.

The main reason for clinical involvement is however not a political one, but the way patient care should be administered. Today a patient expects to see the physician who will perform a treatment in order to develop a trusting relationship.

In 2012 interventional radiology became a primary medical specialty in the United States of America. Since then the best status of interventional radiology is emotionally discussed in Europe. Many interventional radiologists believe that with a specialty status the issues of limited direct patient access, lack of beds and staff would be automatically solved. Dierk Vorwerk described this expectation as “wishful thinking” in 2017 (CVIR 2017; 40:1–2). I agree with this statement. It is the classical chicken-and-egg question: Is clinical care or specialty status first. I would argue that the clinical involvement was first and was the key to achieve specialty status in the United States of America. Therefore I like to motivate all interventional radiologists to start a clinical practice, if they have not done it yet. I suggest starting with an outpatient clinic to consult patients about their disease. This can be done relatively easy and it is a good start to take direct patient responsibilities. Unfortunately clinical services performed by interventional radiologists are not reimbursed by insurance companies in all European countries. This lack of financial compensation can hamper the clinical work within a radiology group. The benefit for patient care are quite obvious and hopefully this will also be seen by the insurance companies.

The Covid-19 pandemic has enhanced the advantages of ambulatory care. Interventional radiology procedures are well suited to be performed in an outpatient setting. Therefore an outpatient lab could be a reasonable next step. Inpatient beds are ultimately desirable to take care of patients after a complex procedure, however this can be difficult in many countries and depends on the individual hospital setting. An interesting solution is to collaborate with another discipline. At our hospital we have merged interventional radiology with vascular surgery to one clinic. I have found this close interdisciplinary collaboration very fruitful not only for staff, but especially for our patients. It is however important that the interventional radiologists truly participates in clinical care! The partnership has to be based on an equal footing. Mahnken et al. (CVIR 2021; 44:1323–1353) have compiled a comprehensive clinical practice manual on clinical skills, patient workflow and practice development which is worth reading.

While I don’t think clinical care can be forced in place by a (sub)specialty status, I believe that interventional should be a recognized subspecialty within radiology across Europe. The trend of continuous specialization will likely continue. The times when a radiologists can do complex procedures, read all sorts of cross-sectional images and mammograms are over. Subspecialty training in radiology is already happening in many places, but so far, it lacks formal recognition in many countries. Because of the clinical aspect of interventional radiology a subspecialty status seems even more important than in other diagnostic areas. Therefore I suggest a subspecialty status of interventional radiology based on at least two year of dedicated training in interventional radiology including clinical skills and the proof of theoretical knowledge with the European Board of Interventional Radiology (EBIR). Having such a clearly defined subspecialty status would increase the quality of patient care. It would also increase the visibility of interventional radiology not only to patients, but also to medical students. This is important because unfortunately interventional radiology is not taught at many universities across Europe and therefore interventional radiology is little known to medical students when they make their decision in which medical field they want to go.

In summary: interventional radiology has become a clinical discipline. I urge all interventional radiologists to get involved in clinical care for their patients: it is worth it. Despite the fact that clinical involvement is not directly dependent on a (sub)specialty status, interventional radiology should be a recognized subspecialty of radiology in order to be visible and to attract medical students.

